# Analysis of a spatial gene expression database for sea anemone *Nematostella vectensis* during early development

**DOI:** 10.1186/s12918-015-0209-4

**Published:** 2015-09-24

**Authors:** Daniel Botman, Fredrik Jansson, Eric Röttinger, Mark Q. Martindale, Johann de Jong, Jaap A. Kaandorp

**Affiliations:** Computational Science, University of Amsterdam, Science Park 904, Amsterdam, The Netherlands; Université Nice Sophia Antipolis, Institute for Research on Cancer and Aging, Nice (IRCAN), UMR 7284, Nice, France; Centre National de la Recherche Scientifique (CNRS), Institute for Research on Cancer and Aging, Nice (IRCAN), UMR 7284, Nice, France; Institut National de la Santé et de la Recherche Médicale (INSERM), Institute for Research on Cancer and Aging, Nice (IRCAN), U1081, Nice, France; Whitney Lab for Marine Bioscience, University of Florida, St. Augustine, FL USA; Computational Cancer Biology Group, Division of Molecular Carcinogenesis, The Netherlands Cancer Institute, Amsterdam, The Netherlands

**Keywords:** Nematostella vectensis, Gene expression quantification, Embryonic development, Cluster analysis, Gene expression database, In Situ Hybridization (ISH)

## Abstract

**Background:**

The spatial distribution of many genes has been visualized during the embryonic development in the starlet sea anemone *Nematostella vectensis* in the last decade. *In situ* hybridization images are available in the Kahi Kai gene expression database, and a method has been developed to quantify spatial gene expression patterns of *N. vectensis*. In this paper, gene expression quantification is performed on a wide range of gene expression patterns from this database and descriptions of observed expression domains are stored in a separate database for further analysis.

**Methods:**

Spatial gene expression from suitable in situ hybridization images has been quantified with the GenExp program. A correlation analysis has been performed on the resulting numerical gene expression profiles for each stage. Based on the correlated clusters of spatial gene expression and detailed descriptions of gene expression domains, various mechanisms for developmental gene expression are proposed.

**Results:**

In the blastula and gastrula stages of development in *N. vectensis*, its continuous sheet of cells is partitioned into correlating gene expression domains. During progressing development, these regions likely correspond to different fates. A statistical analysis shows that genes generally remain expressed during the planula stages in those major regions that they occupy at the end of gastrulation.

**Discussion:**

Observed shifts in gene expression domain boundaries suggest that elongation in the planula stage mainly occurs in the vegetal ring under the influence of the gene Rx. The secondary body axis in N. vectensis is proposed to be determined at the mid blastula transition.

**Conclusions:**

Early gene expression domains in *N. vectensis* appear to maintain a positional order along the primary body axis. Early determination in *N. vectensis* occurs in two stages: expression in broad circles and rings in the blastula is consolidated during gastrulation, and more complex expression patterns appear in the planula within these broad regions. Quantification and comparison of gene expression patterns across a database can generate hypotheses about collective cell movements before these movements are measured directly.

**Electronic supplementary material:**

The online version of this article (doi:10.1186/s12918-015-0209-4) contains supplementary material, which is available to authorized users.

## Background

Spatial gene expression assays can be used as a tool for verifying predicted regulatory interactions between genes and for predicting properties of missing components in a gene regulation network [1, 2]. The largest potential of spatial gene product distribution datasets, is in verifying numerical models of regulatory interaction networks, which has been demonstrated for the embryonic development of fruit fly *Drosophila melanogaster* [3]. Also the formation of digits in early mouse limbs has been replicated with mechanistic models with the help of gene expression patterns [4].

To perform accurate simulations of such processes, the spatial gene expression patterns need to be digitally quantified and formatted to standardized profiles. A procedure for gene expression quantification has been described [5] and applied [6] for organisms with changing morphologies during embryonic development. Similarities among gene expression profiles can provide information about co-expression relationships [7]. Similarity metrics are a common tool for classifying time series expression data to identify correlating dynamics among genes. These similarity measures can identify correlating spatial expression among genes from quantified expression patterns as well. To use quantified gene expression patterns in dynamic simulations, reliable time points for gene expression patterns are required.

For example, in *Drosophila*, the spatial gene expression of *even skipped* (*eve*) has been measured precisely for many time points. The *eve* pattern is employed as a time reference: *eve* is assayed together with the queried gene to establish the development time for the sample [8]. For many other animal models, a time reference gene is not (yet) available and other embryo properties are applied to estimate the time of development. In these cases the subsequent stages of development can be qualitatively identified from the changing embryo morphology. These changes in morphology are caused by division and migration of cells, processes that are absent during the early cleavage cycles of flies.

In the comparative gene expression database Kahi Kai, *in situ* RNA hybridization assays are collected for many marine invertebrates [9] and are classified according to the embryo morphologies. This database thereby allows for an analysis of spatial expression features for all gene entries.

In this study, many genes from the Kahi Kai database are compared at various stages of development, based on their expression in different embryonic regions. First, the majority of *in situ* hybridization images are quantified and the quantified gene expression patterns are collected in a list of digital expression profiles. Stage-specific correlation analyses are performed on these spatial gene expression profiles to discover the embryo’s major division in expression domains.

Second, a subset of genes from the database is listed with a detailed description of the spatial expression in the stages for which data is available. This list provides an overview of the developmental stages with spatial hybridization images for each gene and allows a detailed description of expression properties beyond the general classifications. Progression of spatial expression is compared for subsequent available stages, and the main periods of gene expression dynamics are identified.

A large set of gene expression patterns in the starlet sea anemone *Nematostella vectensis* is analyzed. The determination of the secondary axis in *N. vectensis* is one aspect of gene expression that requires spatial localization. The database contains various genes that are expressed along this axis.

**Fig. 1:**
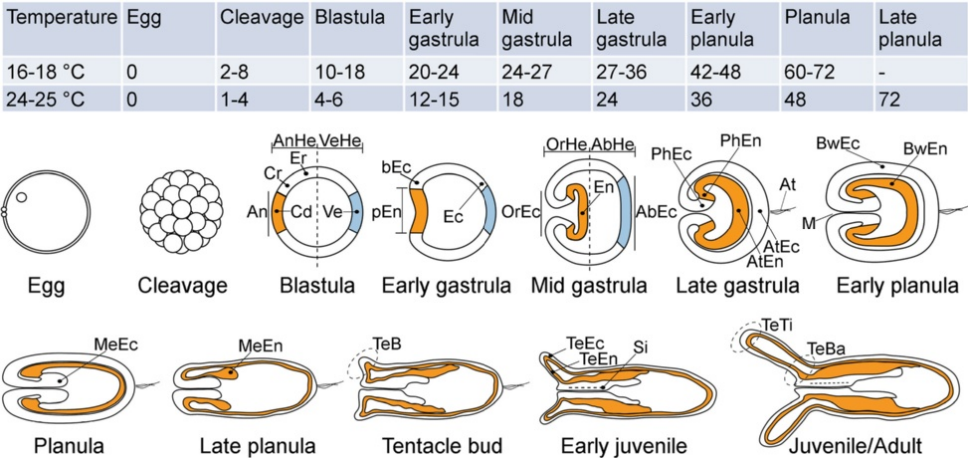
Progressing embryo morphology during *N. vectensis* development. The table estimates the time of development at two different temperatures for the stages until the late planula. Table entries indicate the hours after fertilization derived from [11, 18, 21]. The annotations in the schematic morphologies are guidelines for researchers to describe expression domains in their hybridization images. AnHe = animal hemisphere, VeHe = vegetal hemisphere, An = animal pole, Cd = central domain, Cr = central ring, Er = external ring, Ve = vegetal pole, pEn = presumptive endoderm, bEc = blastoporal ectoderm, Ec = ectoderm, OrHe = oral hemisphere, AbHe = aboral hemisphere, OrEc = oral ectoderm, En = endoderm, AbEc = aboral ectoderm, PhEc = pharyngeal ectoderm, PhEn = pharyngeal endoderm, AtEn = apical tuft endoderm, AtEc = apical tuft ectoderm, At = apical tuft, M = mouth, BwEc = body wall ectoderm, BwEn = body wall endoderm, MeEc = mesentery ectoderm, MeEn = mesentery endoderm, TeB = tentacle bud, TeEc = tentacle ectoderm, TeEn = tentacle endoderm, Si = siphonoglyph, TeTi = tentacle tip, TeBa = tentacle base (The original nomenclature in [9] has been adapted to the more detailed denotations for the blastula stage in [15].)

The change in *N. vectensis* morphology during development is schematically displayed in Fig. 1. The nucleus in the egg is located at the future oral pole, which means that the primary (oral-aboral) axis is already determined before fertilization [10, 11]. The determination of the secondary axis, which is defined by the location of the syphonoglyph, is unclear. The first structures that appear along this axis are the primary mesenteries, but differential gene expression is already observed during gastrulation [12, 13]. Based on the early symmetry break in various gene expression patterns and on early *N. vectensis* morphogenesis, a mechanism is proposed for secondary axis determination. Spatial gene expression patterns in early stages of development are necessary to study the determination and formation of the secondary axis.

We analyzed spatial gene expression patterns in various stages of development in *N. vectensis*. Changes in these patterns are due to gene expression dynamics within stationary cells or due to migrating cells that retain their gene expression state. Fate mapping experiments can conclusively determine migratory behavior, but these data are not available for *N. vectensis*. Our solution to deal with this lack of fate map data is the assumption that the expression state in migrating cells likely remains unchanged for many genes. In this fashion, we estimate major cell movements based on the spatial gene expression data, which are available.

In conclusion we demonstrate the application of correlation analysis to quantified spatial gene expression patterns in order to identify co-expressed spatial domains.

A possible application of these correlation matrices is the selection of gene clusters for regulatory experiments, functional studies [14] and computational gene regulation network models [6], because co-expressed genes often share regulators or biological functions.

## Methods

**Fig. 2:**
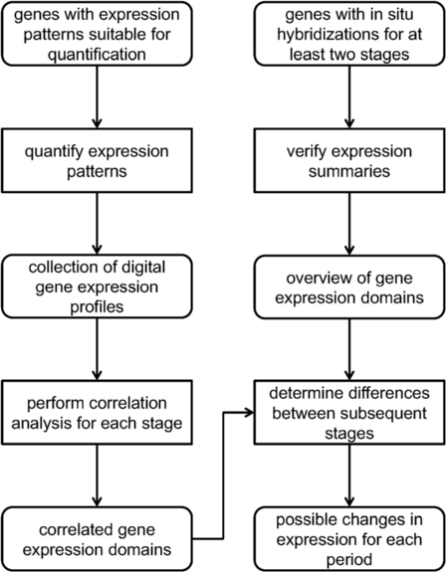
Workflow overview. The information stored in the Kahi Kai gene expression database has been processed into convenient formats for two partly overlapping sets of genes. These processing methods and the methods used for additional analyses are described in the text. While this workflow may seem to converge to a single final result, all intermediate results can be explored for multiple purposes

The order of application for the described methods is displayed in the diagram in Fig. 2. Note that the intermediate results provide new information on their own and can be subjected to additional analyses.

### Cluster analysis of quantified *in situ* hybridizations

**Fig. 3:**
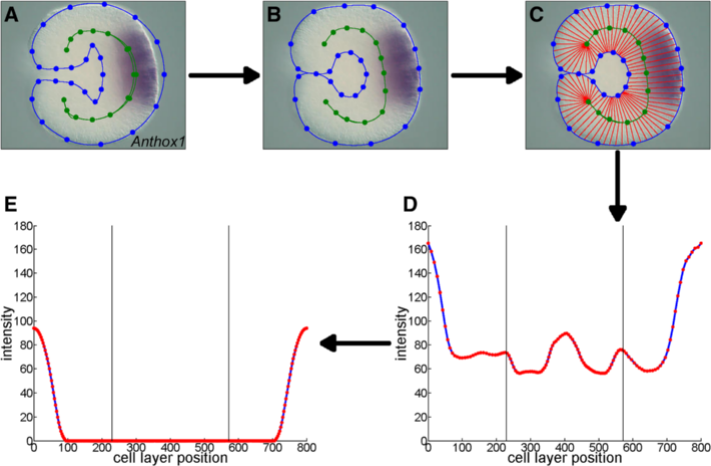
Gene expression quantification pipeline. A digital morphology is overlaid with the gene expression image (**a**) and the points are manually dragged over the embryo’s cell layer (**b**). After the cell layer is decomposed into segments (**c**) and the intensity is plotted as a function of cell layer position (**d**), the profile is manually edited to correct for artifacts (**e**)

The genes listed in the Kahi Kai database for *N. vectensis* are screened for useful expression patterns. For one-dimensional quantification, suitable genes are genes that display cylindrical expression in broad domains up to the late planula stage. Other genes, such as those that are expressed on the syphonoglyph side only or in individual cells, require a two-dimensional or three-dimensional quantification method for a complete description. *In situ* hybridization images are imported into GenExp, a MATLAB interface designed to extract and quantify gene expression patterns [5]. A continuous series of digital morphologies has been derived from a confocal microscopy study on *N. vectensis* gastrulation. A digital morphology is selected and overlaid with the hybridization image (Fig. 3a). To get a correct alignment, the points of the digital morphology are dragged over the observed cell layer boundaries (Fig. 3b). The cell layer is divided into segments with edges between the inner and outer cell layer boundaries (Fig. 3c). The color intensities of the pixels within each segment are averaged and plotted as a function of the segment’s position on the cell layer (Fig. 3d). This plot is edited to compensate for artifacts from the environment, annotations and imperfections in the segmentation (Fig. 3e). The edited plot is interpolated at a hundred equidistant points and the intensity is scaled to unity to arrive at a standardized expression profile. The standardized profiles are ordered in seven groups from blastula to late planula, based on the development label of the source images. The profiles within each group are clustered with average linkage and Pearson correlation distance. The groups are divided into two main clusters, or into three clusters if the second split reduces the largest branch. The profiles within these main clusters are displayed in combined plots. Profiles in any apparent subclusters are plotted together as well.

### Overview and analysis of expression summaries

**Fig. 4:**
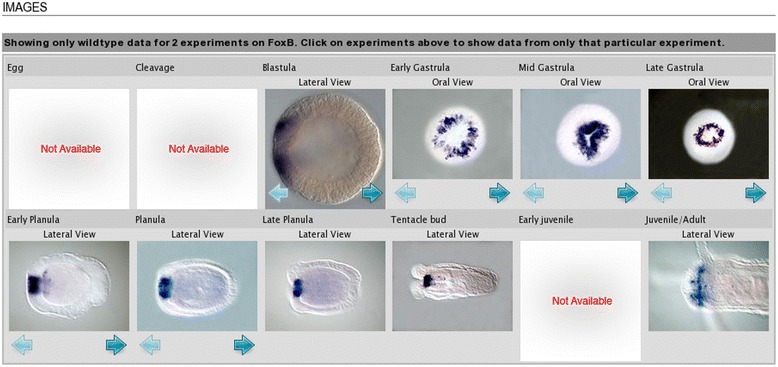
Hybridization images for *FoxB* in the Kahi Kai database. If multiple images are available for a developmental stage, these are accessed with the blue arrow buttons

**Fig. 5:**
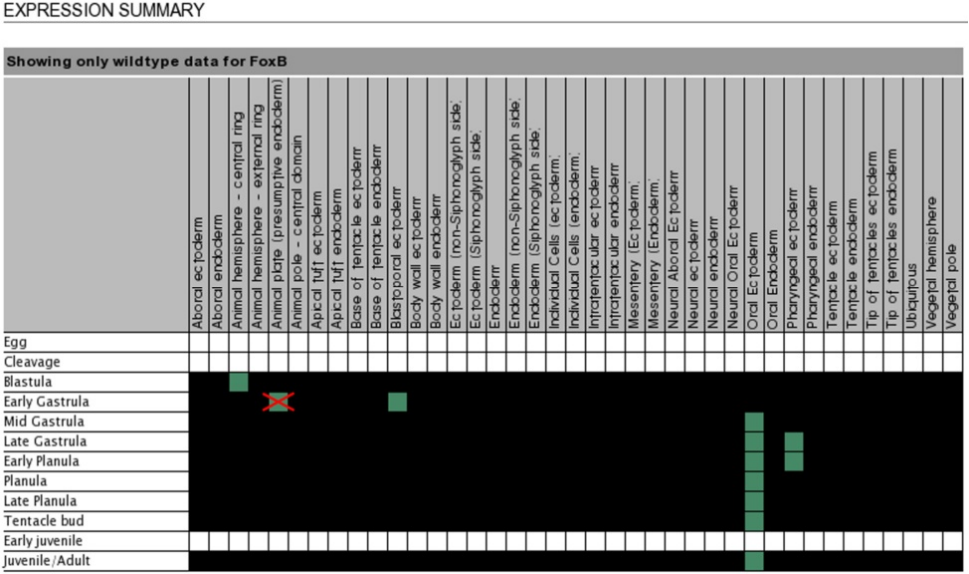
Expression summary for *FoxB* in the Kahi Kai database. The indicated expression domains are derived from the available *in situ* RNA hybridizations (some hybridization images are displayed in Fig. 4). Expression in the presumptive endoderm at the early gastrula is incorrect

A list of all genes in the Kahi Kai database with *in situs* available from the blastula to the late planula stage was retrieved with the built-in search tool. From this list, those genes were selected with images available in at least two different stages of development. The selected genes are listed in a database table, with descriptions of the expression patterns in available stages. The descriptions are derived from the Kahi Kai expression summary matrix, while correcting possible inconsistencies with the *in situs* (illustrated for the gene *FoxB* in Figs. 4 and 5 as an example). Expression in the endoderm wall and ectoderm wall is specified in more detail. If a gene expression pattern exhibits noncylindrical symmetry, this is briefly indicated.

All possible pairwise combinations of developmental stages are listed in a spreadsheet, and the expression pattern descriptions from the database table are inserted for subsequent available stages. All combinations with identical expression domains in both the first and the second stage are added up. The extent to which the expression pattern has changed is indicated in a separate column. The instances in which a pattern has remained within the same major region (minor change), shifted across major regions (major change) or vanished are catalogued for genes that start in a single major domain. For each major domain, all possible stages in which a pattern can display a minor change, display a major change or vanish are counted. The relative occurrences of these events in each stage are derived from their counts. The instances that a pattern has displayed minor or major changes with respect to the major expression regions are registered for the complete set of available genes as well, along with their possible first appearance and disappearance.

## Results

### Cluster analysis of quantified *in situ* hybridizations

**Fig. 6:**
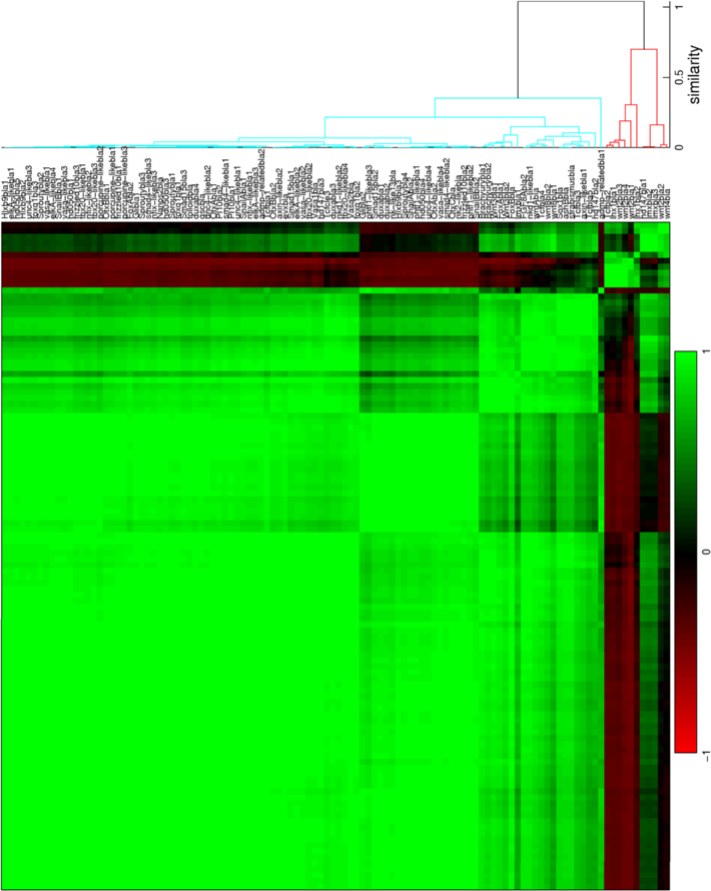
Hierarchical clustering of gene expression profiles in the blastula stage. Tcfcle3 is labeled as cleavage, but with a large blastocoel the sample is suitable for quantification. The dendrogram is cut off at a similarity (1 minus the correlation coefficient) of 0.7. For all clusterings, Pearson correlation is used as the distance metric and unweighted average is used for linking

**Fig. 7:**
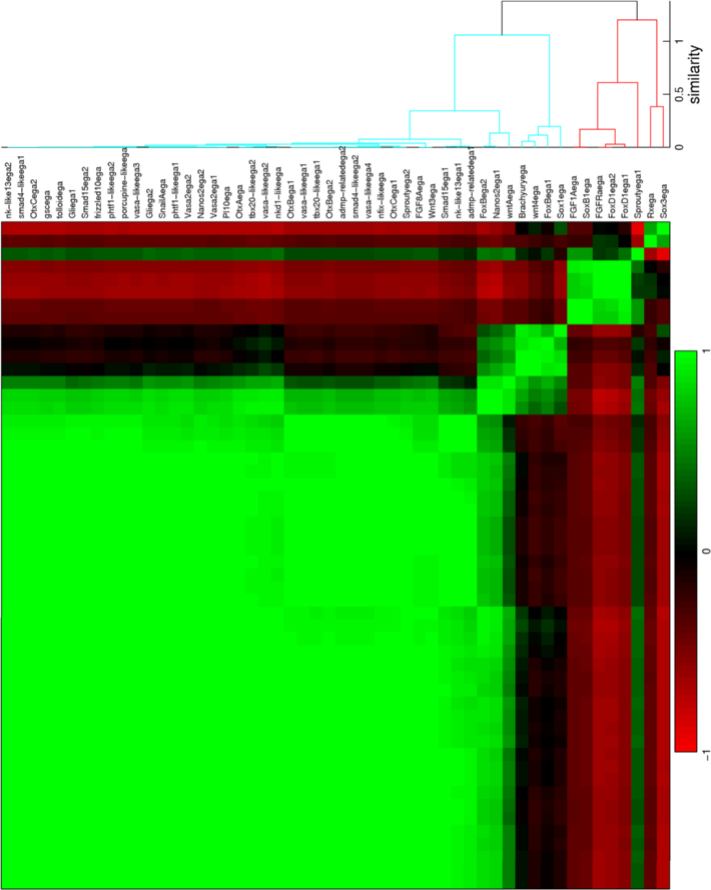
Hierarchical clustering of gene expression profiles in the early gastrula stage. The dendrogram is cut off at a similarity of 1.3

**Fig. 8:**
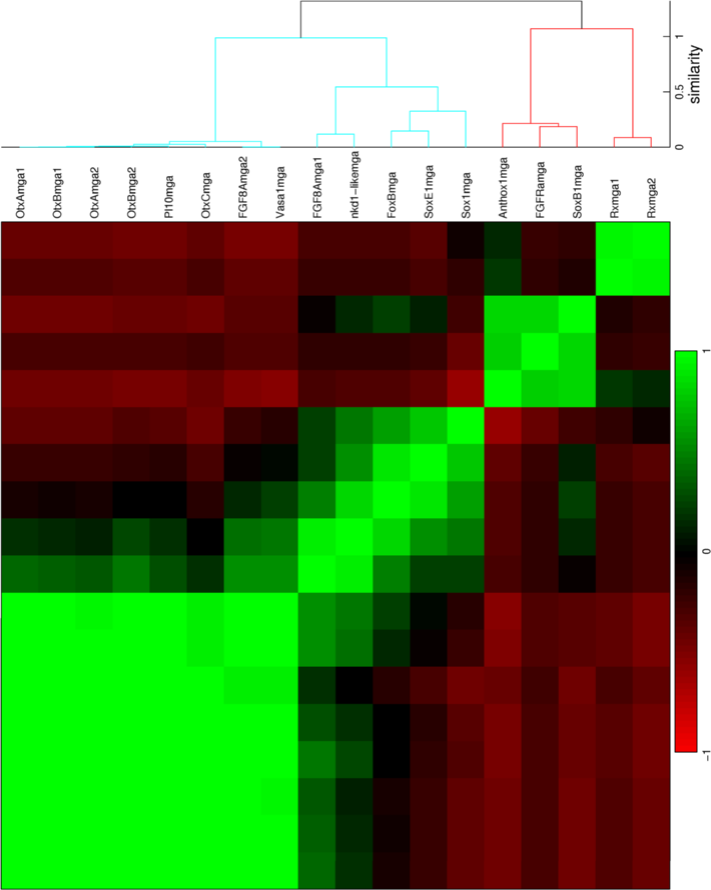
Hierarchical clustering of gene expression profiles in the mid gastrula stage. The dendrogram is cut off at a similarity of 1.1

**Fig. 9:**
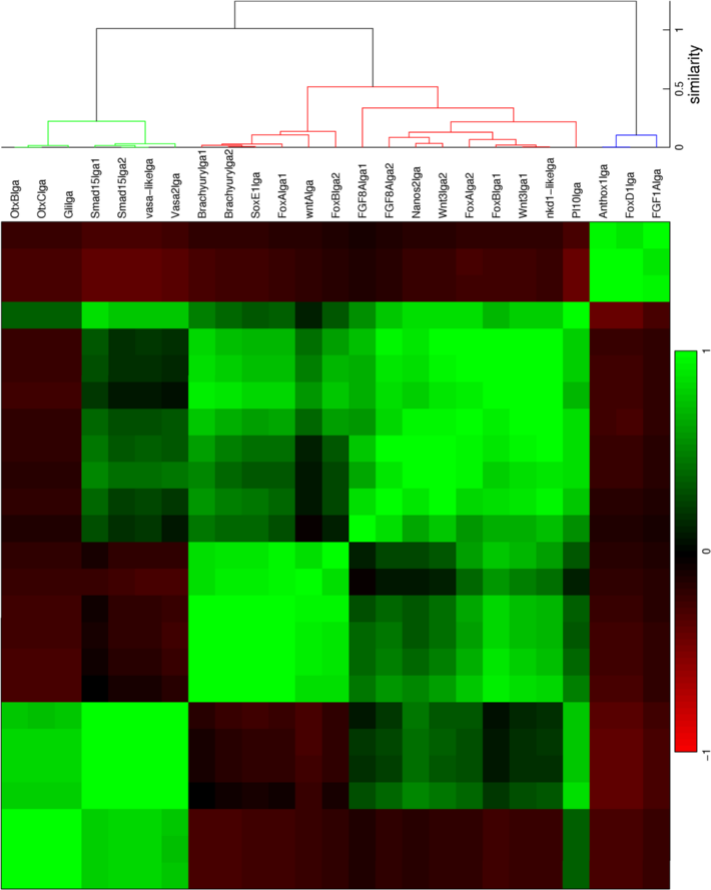
Hierarchical clustering of gene expression profiles in the late gastrula stage. The dendrogram is cut off at a similarity of 0.6

**Fig. 10:**
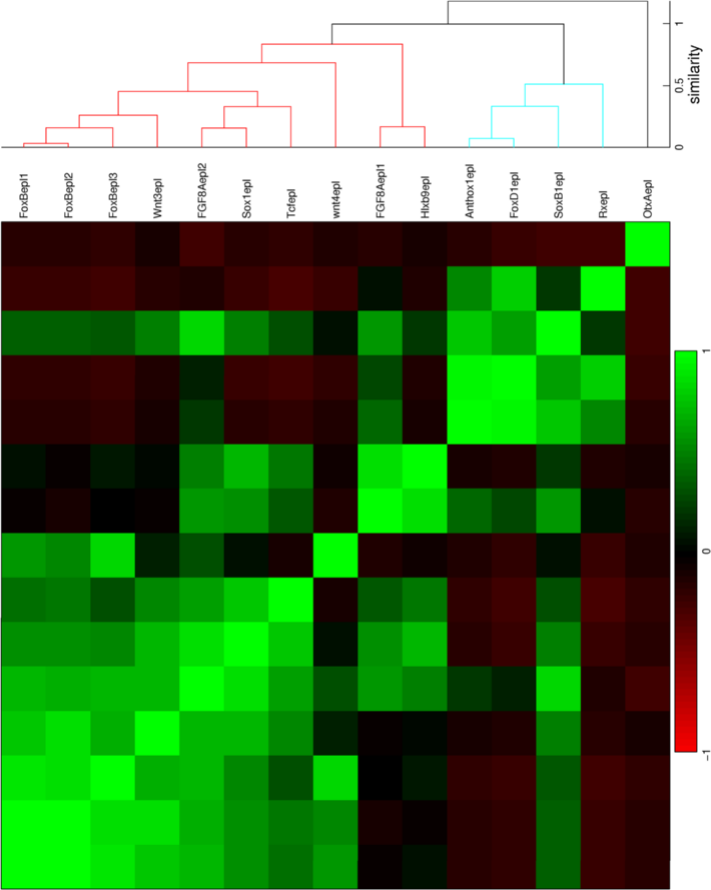
Hierarchical clustering of gene expression profiles in the early planula stage. The dendrogram is cut off at a similarity of 0.9

**Fig. 11:**
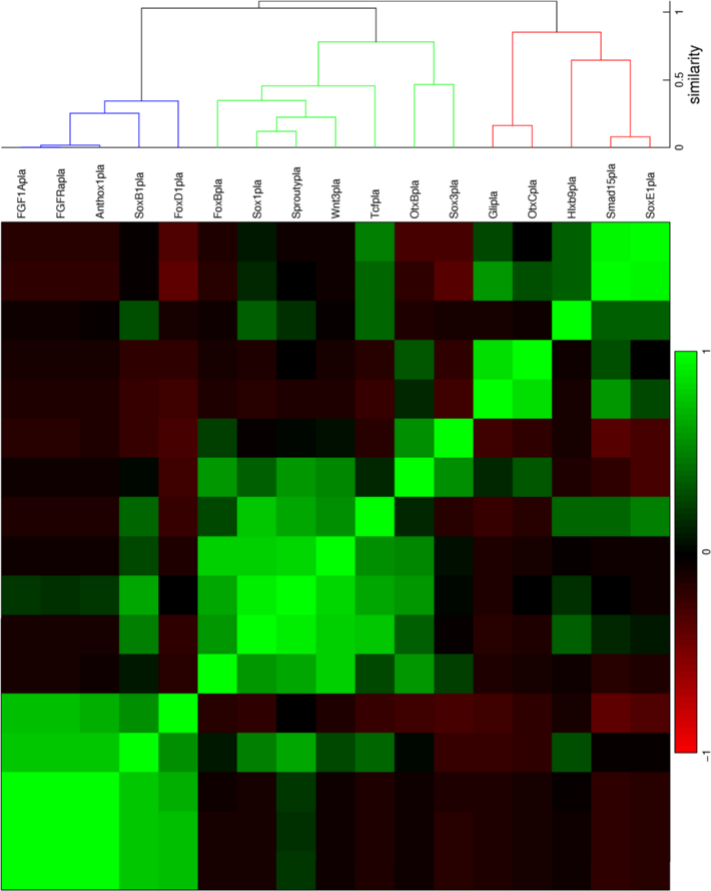
Hierarchical clustering of gene expression profiles in the planula stage. The dendrogram is cut off at a similarity of 0.9

**Fig. 12:**
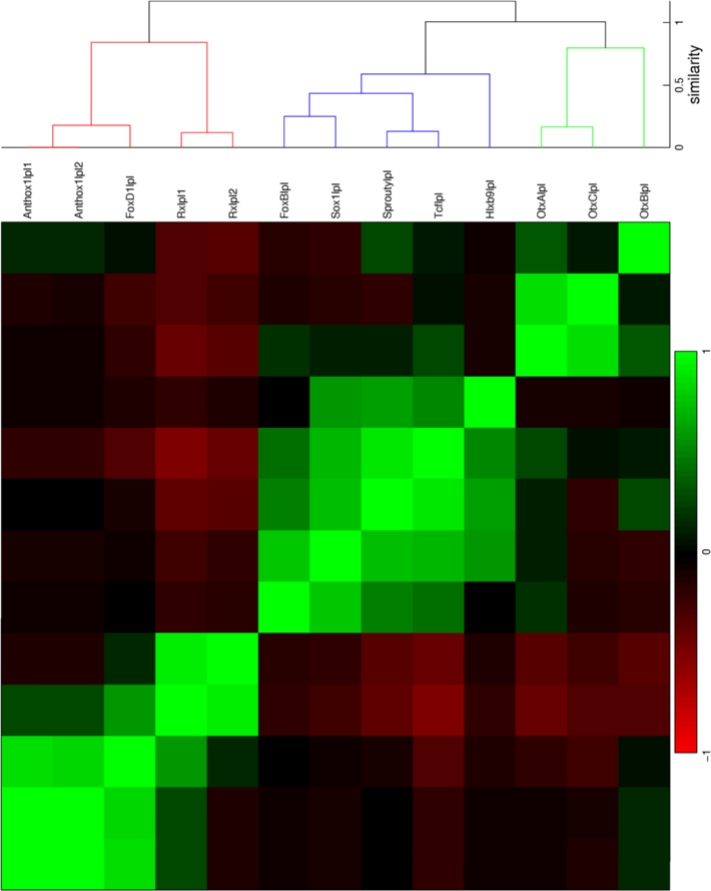
Hierarchical clustering of gene expression profiles in the late planula stage. The dendrogram is cut off at a similarity of 0.9

**Fig. 13:**
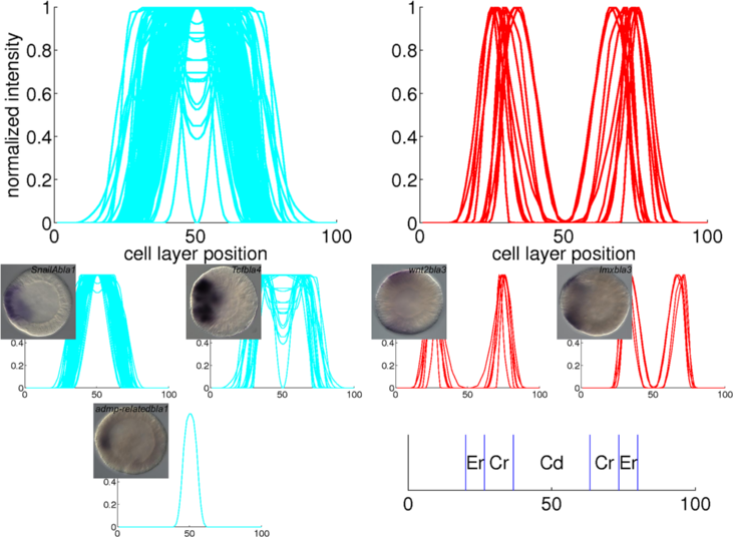
Combined plots of quantified gene expression patterns in the blastula stage. The main clusters from Fig. 6 are plotted in large diagrams. The small diagrams are subclusters within the large plot above them. The insets are *in situ* hybridizations from which a profile in the subcluster is derived. The expression domains that arise from the clustering are: central domain (Cd), central ring (Cr) and external ring (Er)

**Fig. 14:**
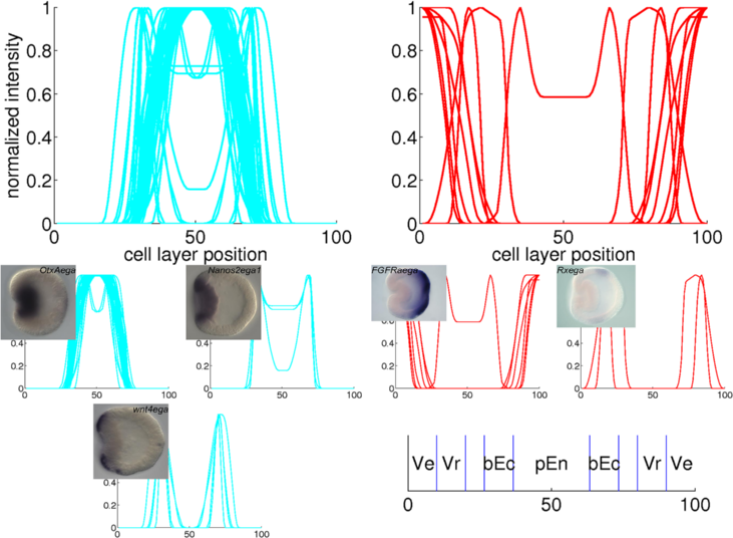
Combined plots of quantified gene expression patterns in the early gastrula stage. The main clusters from Fig. 7 are plotted in large diagrams. The small diagrams are subclusters within the large plot above them. The insets are *in situ* hybridizations from which a profile in the subcluster is derived. The expression domains that arise from the clustering are: presumptive endoderm (pEn), blastoporal ectoderm (bEc), vegetal ring (Vr) and vegetal pole (Ve)

**Fig. 15:**
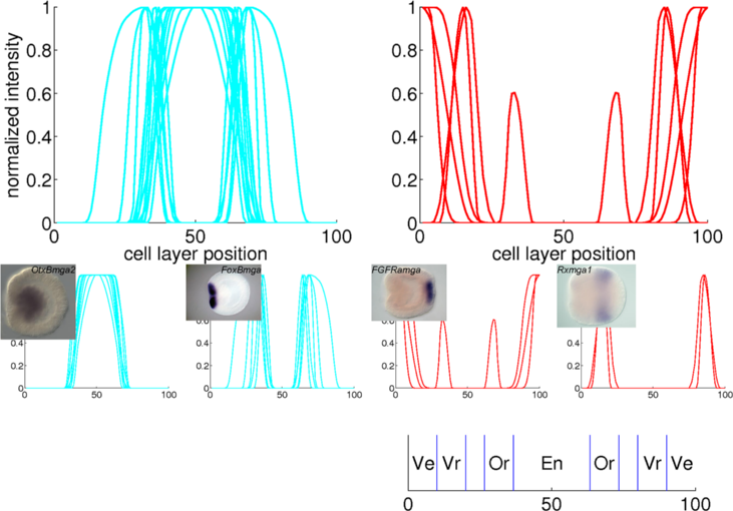
Combined plots of quantified gene expression patterns in the mid gastrula stage. The main clusters from Fig. 8 are plotted in large diagrams. The small diagrams are subclusters within the large plot above them. The insets are *in situ* hybridizations from which a profile in the subcluster is derived. The expression domains that arise from the clustering are: endoderm (En), oral pole (Or), vegetal ring (Vr) and vegetal pole (Ve)

**Fig. 16:**
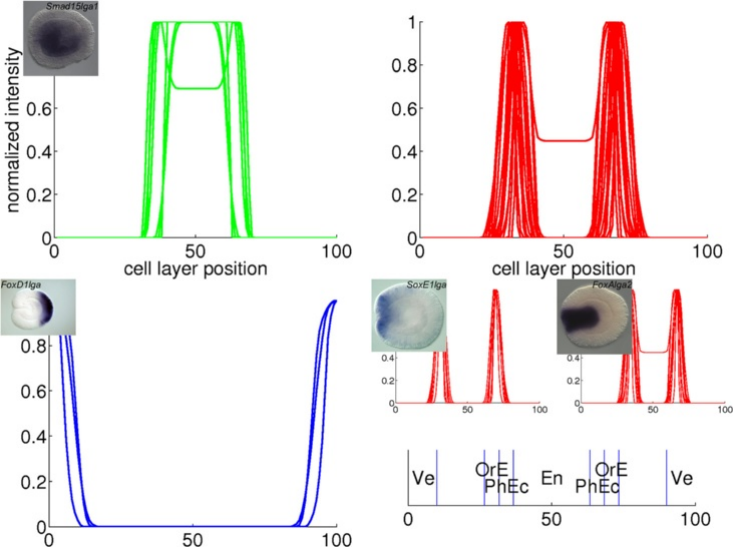
Combined plots of quantified gene expression patterns in the late gastrula stage. The main clusters from Fig. 9 are plotted in large diagrams. The small diagrams are subclusters within the large plot above them. The insets are *in situ* hybridizations from which a profile in the (sub)cluster is derived. The expression domains that arise from the clustering are: endoderm (En), pharyngeal ectoderm (PhEc), oral end (OrE) and vegetal pole (Ve)

**Fig. 17:**
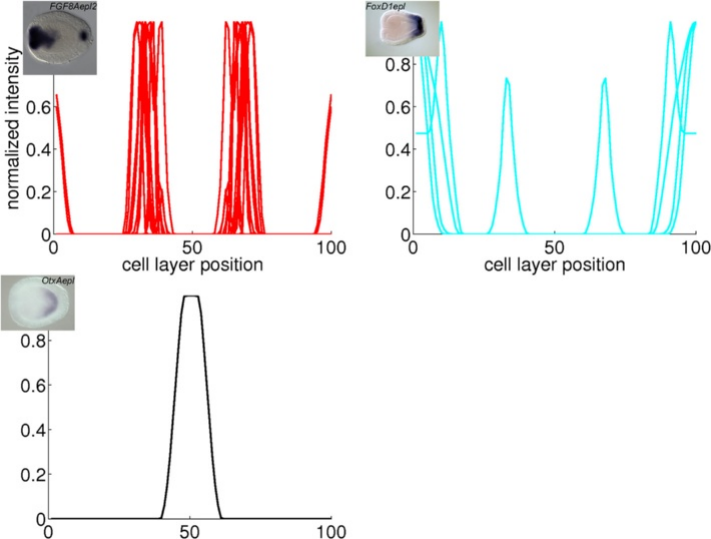
Combined plots of quantified gene expression patterns in the early planula stage. The plots represent the clusters in Fig. 10. The insets are *in situ* hybridizations from which a profile in the cluster is derived

**Fig. 18:**
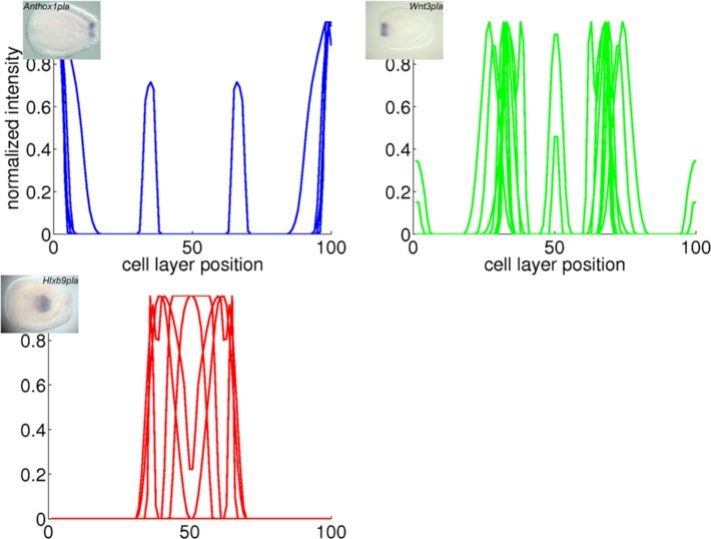
Combined plots of quantified gene expression patterns in the planula stage. The plots represent the clusters in Fig. 11. The insets are *in situ* hybridizations from which a profile in the cluster is derived

**Fig. 19:**
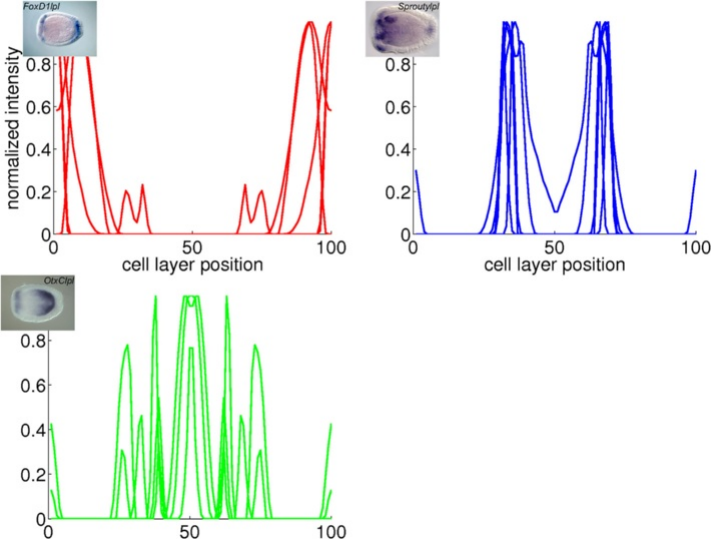
Combined plots of quantified gene expression patterns in the late planula stage. The plots represent the clusters in Fig. 12. The insets are *in situ* hybridizations from which a profile in the cluster is derived

**Fig. 20:**
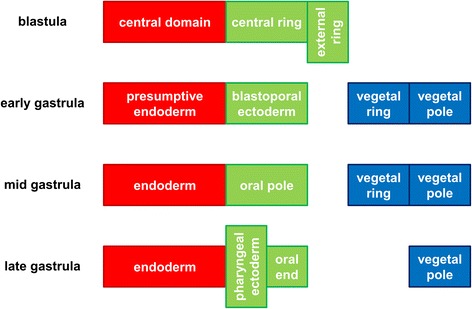
Overview of gene expression regions at various stages. The clusters and subclusters of correlating standardized expression profiles have been divided in three major regions: central domain/endoderm (*red*), central ring/external ring/oral ectoderm (*green*) and vegetal hemisphere/aboral ectoderm (*blue*)

For all seven stages of development from blastula to late planula, *in situs* of suitable genes have been quantified. The expression profiles are provided in Additional file 1 in MATLAB format. The quantified patterns are ordered in dendrograms and correlation matrices. The number of analyzed patterns from each stage are: 112 from the blastula (Fig. 6); 52 from the early gastrula (Fig. 7); 18 from the mid gastrula (Fig. 8); 25 from the late gastrula (Fig. 9); 15 from the early planula (Fig. 10); 17 from the planula (Fig. 11) and 13 from the late planula (Fig. 12). From the correlation matrices, major and minor blocks are identified. The profiles in these blocks are combined in separate plots for the blastula (Fig. 13), early gastrula (Fig. 14), mid gastrula (Fig. 15), late gastrula (Fig. 16), early planula (Fig. 17), planula (Fig. 18) and late planula (Fig. 19) stages. Correlating expression domains from the blastula to the late gastrula stages are summarized in Fig. 20.

For the blastula stage, gene expression is present in the central domain in the 101 profiles in the blue cluster, while expression is excluded from the central domain in the 11 profiles in the red cluster. From the correlation matrix in Fig. 6, two smaller blocks with strong correlation and an isolated sample are identified within the blue cluster. The profiles within each subcluster are combined in three small plots below the major blue cluster in Fig. 13. The first subcluster contains profiles with gene expression limited to the central domain, while gene expression in the second subcluster is extended to the central ring. The isolated profile displays gene expression in a narrow spot within the central domain. The red cluster in the correlation matrix contains two smaller blocks as well. These subclusters, the small plots below the major red cluster in Fig. 13, seem to separate the central ring and the external ring. An earlier study of whole mount *in situ* hybridizations in the *N. vectensis* blastula distinguished four co-expression domains in the animal hemisphere [15]. The present cluster analysis confirms the existence of these four domains (central domain, central domain + central ring, central ring and external ring).

For the early gastrula stage, gene expression is absent in the aboral ectoderm in the 44 profiles in the blue cluster, while this region is included in the expression patterns of the 8 profiles in the red cluster. The blue cluster of the correlation matrix (Fig. 7) contains three smaller blocks; two outer blocks are clearly separated, while the middle block is positively correlated to both other blocks. The profiles within each subcluster are combined in three small plots below the major blue cluster in Fig. 14. Expression in the first and third subclusters is limited to the presumptive endoderm and blastoporal ectoderm, respectively. Expression in the second subcluster covers both regions. The red cluster of the correlation matrix contains two smaller blocks; these subclusters are plotted in Fig. 14 below the major red cluster. The first subcluster exhibits expression at the vegetal pole, while genes in the second subcluster are expressed in a ring around the vegetal pole.

For the mid gastrula stage, the blue cluster consists of 13 profiles with expression in the blastopore, while gene expression appears in the aboral ectoderm in the 5 profiles in the red cluster. The blue cluster of the correlation matrix in Fig. 8 contains a block with strongly correlated profiles, while the remaining profiles are correlated somewhat weaker. The first subcluster represents endodermal expression, while expression in the second subcluster is in the oral pole at various ranges from the center. The profiles of both subclusters are displayed in Fig. 15 below the major blue cluster. The red cluster of the correlation matrix contains two blocks with strongly correlated profiles. The profiles in these blocks, combined in two small plots below the major red cluster in Fig. 15, represent gene expression at the vegetal pole and in a ring around this pole, respectively.

For the late gastrula stage, the green cluster represents endodermal expression, the red and blue clusters represent ectodermal expression in the oral and aboral pole, respectively. Two smaller blocks are visible within the red cluster; the profiles in these subclusters are displayed in two small plots in Fig. 16. Gene expression in the first subcluster is limited to the oral end, while expression in the second subcluster is extended inwards to the pharynx.

For the early planula stage, the three clusters represent the oral pole, the aboral ectoderm and the aboral endoderm. The profiles in these clusters are plotted in red, blue and black in Fig. 17, respectively.

For the planula stage, the characteristic expression domains in each of the three clusters are the aboral pole ectoderm, the oral pole ectoderm and the endoderm, respectively. The profiles in these clusters are collected in the blue, green and red plots in Fig. 18, respectively.

For the late planula stage, the first two clusters show expression in the aboral and oral ectoderm, respectively, while the profiles in the third cluster show expression in multiple locations. The clusters are displayed in the red, blue and green plots in Fig. 19, respectively.

### Overview and analysis of expression summaries

From the Kahi Kai gene expression database, 73 genes have hybridization images available for at least two stages from blastula to late planula. These genes are listed with descriptions of their expression in a Microsoft Access database sheet (Additional file 2). Counts of pairwise expression domains are listed in a Microsoft Excel spreadsheet (Additional file 3). The central domain/endoderm, central ring/external ring/oral ectoderm and vegetal hemisphere/aboral ectoderm are selected as major expression regions.

The changes or lack of change in expression patterns starting in a single major expression region are shown in Table 1 (central domain/endoderm), Table 2 (central ring/external ring/oral ectoderm) and Table 3 (vegetal hemisphere/aboral ectoderm). Sums of blocks from these matrices are displayed in Table 4, Table 5 and Table 6, respectively.

**Table 1:** Gene expression behavior with initial expression limited to the central domain/endoderm

	Initial stage	Blastula	Early gastrula	Mid gastrula	Late gastrula	Early planula	Planula
final stage							
early gastrula		(21:1:1)					
mid gastrula		**(1:0:0)**	**(4:2:0)**				
late gastrula		**(0:0:0)**	**(4:1:0)**	(3:0:0)			
early planula		**(0:1:0)**	**(0:0:0)**	(1:0:0)	(0:0:0)		
planula		**(0:0:0)**	**(0:0:0)**	(0:0:0)	(4:1:0)	(7:0:0)	
late planula		**(0:0:0)**	**(0:0:0)**	(0:0:0)	(0:0:0)	(0:1:0)	(9:0:0)

Entries from the pairwise gene expression spreadsheet (Additional file 3) that start with expression only in the central domain/endoderm are included in this table. For all combinations of stages, the modes of progression of gene expression (minor change:major change:vanished) are counted. The combinations in bold numbers include the period from the early gastrula to the mid gastrula

**Table 2:** Gene expression behavior with initial expression limited to the central ring/external ring/oral ectoderm

	Initial stage	Blastula	Early gastrula	Mid gastrula	Late gastrula	Early planula	Planula
Final stage							
Early gastrula		(8:0:0)					
Mid gastrula		(0:0:0)	(5:0:0)				
Late gastrula		(1:0:0)	(2:2:0)	(8:0:0)			
Early planula		(0:1:0)	(1:0:0)	(1:0:0)	(4:1:0)		
Planula		(0:0:0)	(0:0:0)	(0:0:0)	(2:1:0)	(4:0:1)	
Late planula		(0:0:0)	(0:0:0)	(0:0:0)	(0:0:0)	(1:0:0)	(3:0:0)

Entries from the pairwise gene expression spreadsheet (Additional file 3) that start with expression only in the central ring/external ring/oral ectoderm are included in this table. For all combinations of stages, the modes of progression of gene expression (minor change:major change:vanished) are counted

**Table 3:** Gene expression behavior with initial expression limited to the vegetal hemisphere/aboral ectoderm

	Initial stage	Blastula	Early gastrula	Mid gastrula	Late gastrula	Early planula	Planula
Final stage							
Early gastrula		(0:0:0)					
Mid gastrula		(0:0:0)	(2:1:0)				
Late gastrula		(0:0:0)	(2:0:0)	(1:0:0)			
Early planula		(0:0:0)	(0:0:0)	(1:0:0)	(3:0:0)		
Planula		(0:0:0)	(0:1:0)	(1:0:0)	(1:0:0)	(2:0:0)	
Late planula		(0:0:0)	(0:0:0)	(0:0:0)	(0:0:0)	(1:0:0)	(1:1:0)

For pairs of subsequent stages, the modes of progression of gene expression (minor change:major change:vanished) are counted

**Table 4:** Sums of possible periods for gene expression behavior in the central domain/endoderm

Two-stage period	Possible counts	Percentages
Blastula-early gastrula	(22:2:1)	(88:8:4)
Early gastrula-mid gastrula	**(9:4:0)**	(69:31:0)
Mid gastrula-late gastrula	(8:2:0)	(80:20:0)
Late gastrula-early planula	(5:2:0)	(71:29:0)
Early planula-planula	(11:2:0)	(85:15:0)
Planula-late planula	(9:1:0)	(90:10:0)

For each period, the possible changes in gene expression (minor change:major change:vanished) are added in the second column and expressed as percentages in the third column. As an example, the period from the early gastrula to the mid gastrula (bold numbers) is included in the combinations of stages highlighted in Table 1

**Table 5:** Sums of possible periods for gene expression behavior in the central ring/external ring/oral ectoderm

Two-stage period	Possible counts	Percentages
Blastula-early gastrula	(9:1:0)	(90:10:0)
Early gastrula-mid gastrula	(9:3:0)	(75:25:0)
Mid gastrula-late gastrula	(13:3:0)	(81:19:0)
Late gastrula-early planula	(8:3:0)	(73:27:0)
Early planula-planula	(7:1:1)	(78:11:11)
Planula-late planula	(4:0:0)	(100:0:0)

For each period, the possible changes in gene expression (minor change:major change:vanished) are added in the second column and expressed as percentages in the third column

**Table 6:** Sums of possible periods for gene expression behavior in the vegetal hemisphere/aboral ectoderm

Two-stage period	Possible counts	Percentages
Blastula-early gastrula	(0:0:0)	(0:0:0)
Early gastrula-mid gastrula	(4:2:0)	(67:33:0)
Mid gastrula-late gastrula	(5:1:0)	(83:17:0)
Late gastrula-early planula	(6:1:0)	(86:14:0)
Early planula-planula	(5:1:0)	(83:17:0)
Planula-late planula	(2:1:0)	(67:33:0)

For each period, the possible changes in gene expression (minor change:major change:vanished) are added in the second column and expressed as percentages in the third column

A total of 25 genes for which *in situs* are available in the blastula stage are exclusively expressed in the central domain. From these genes, 22 are expressed in the endoderm in the next stage with available *in situs*. Expression of 2 genes has changed beyond the endoderm in the next available stage and 1 gene is no longer expressed at all. This means that 88 % of the genes expressed only in the central domain in the blastula is subsequently expressed only in the endoderm. From all combinations of subsequent available stages that include the early gastrula-mid gastrula period (highlighted in Table 4), 13 genes are initially only expressed in the central domain/endoderm. From these genes, 4 expression patterns have changed beyond the endoderm, which is 31 %. Likewise, out of the 10 genes initially limited to the central domain/endoderm, the major change of 2 (20 %) could possibly occur in the mid gastrula-late gastrula period. Out of the 7 genes expressed between the late gastrula and early planula, 2 (29 %) could have changed beyond the endoderm during this interval.

Out of the 10 genes expressed only in the central ring or external ring in the blastula, 9 (90 %) are subsequently expressed only in the oral ectoderm.

No blastula *in situs* are available for genes expressed in the vegetal hemisphere. Out of the 6 genes expressed only in the early gastrula vegetal hemisphere, 4 (67 %) are subsequently expressed only in the aboral ectoderm.

The changes or lack of change in expression in all regions are shown in Table 7. Sums of blocks from this matrix are displayed in Table 8. Out of all 41 genes with images stored in the blastula stage, 31 (76 %) display expression in the next available stage in the same major region(s). The added percentages for major changes and first appearance of gene expression are highest in the early gastrula to early planula stages (*n* = 214, *p* = 0.03, two-tail Fisher’s exact test).

**Table 7:** Gene expression behavior for all regions combined

	Initial stage	Blastula	Early gastrula	Mid gastrula	Late gastrula	Early planula	Planula
Final stage							
Early gastrula		(29:3:1:1:1)					
Mid gastrula		(1:0:2:0:0)	(12:3:0:0:1)				
Late gastrula		(1:0:0:0:0)	(8:3:2:0:0)	(12:0:1:0:0)			
Early planula		(0:2:0:0:0)	(1:0:1:0:0)	(4:1:4:0:0)	(8:2:1:0:0)		
Planula		(0:0:0:0:0)	(0:3:3:0:0)	(2:0:3:0:0)	(7:2:2:0:0)	(17:0:0:1:0)	
Late planula		(0:0:0:0:0)	(0:0:0:0:0)	(0:2:1:0:0)	(0:0:0:0:0)	(2:1:0:0:0)	(16:2:0:0:0)

All entries from the pairwise gene expression spreadsheet (Additional file 3) are included in this table. For all combinations of stages, the modes of progression of gene expression (minor change:major change:appeared:vanished:none) are counted

**Table 8:** Sums of possible periods for gene expression behavior in all regions

Two-stage period	Possible counts	Percentages
Blastula-early gastrula	(31:5:3:1:1)	(76:12:7:2:2)
Early gastrula-mid gastrula	(23:11:8:0:1)	(53:26:19:0:2)
Mid gastrula-late gastrula	(28:11:15:0:0)	(52:20:28:0:0)
Late gastrula-early planula	(22:12:15:0:0)	(45:24:31:0:0)
Early planula-planula	(28:8:9:1:0)	(61:17:20:2:0)
Planula-late planula	(18:5:1:0:0)	(75:21:4:0:0)

For each period, the possible changes in gene expression (minor change:major change:appeared:vanished:none) are added in the second column and expressed as percentages in the third column

The latter trend was also observed for the subsets of genes initially expressed exclusively in the endoderm and in the oral ectoderm, respectively. This could not be statistically confirmed though (*p* = 0.31 and *p* = 0.42, respectively), due to lower numbers (*n* = 53 and *n* = 51, respectively). The data points for genes expressed in the aboral ectoderm are too few (*n* = 28) to observe any trend.

## Discussion

A gene with expression profiles from multiple samples at one stage can belong to more than one cluster. This may be due to the noise in gene expression among individuals. Another possible explanation is that the expression patterns are different across individual *in situs* at the beginning and at the end of a developmental stage. This change could be caused by cells migrating from one region to another, or by dynamic regulatory interactions. In the current study, this issue is handled by performing cluster analyses to all expression profiles for all genes within a broad time window instead of analyzing blurred averages of each gene. A solution to this uncertainty would be an increased time resolution for the expression profiles, resulting in more precise regions for narrower time windows. In *Drosophila* embryos, the definition of narrow time classes allowed the observation of significant domain shifts [4]. In the sea urchin, precise timing resulted in a sequence of spatial regulatory states [16].

**Fig. 21:**
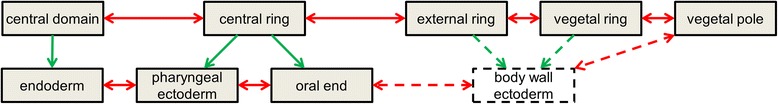
Proposed interactions between gene clusters in various expression regions. Early expression clusters activate or develop into later expression clusters in corresponding regions (*green arrows*). Neighboring expression clusters inhibit each other (*red arrows*). The blastula domains developing into the body wall ectoderm region are derived from a single gene expression pattern (see text). For this reason, the proposed interactions with the body wall ectoderm are indicated with dashed arrows

Central domain expression is generally persistent in the endoderm, while expression in the central and external rings is often limited to the oral ectoderm during and after gastrulation. The first entry in Table 1 shows that the early gastrula expression pattern is known for 23 genes that are expressed exclusively in the central domain in the blastula stage. Out of these 23 genes, 21 are expressed in the presumptive endoderm in the early gastrula stage. Similarly, all 8 genes expressed exclusively in the central or external ring in the blastula with known expression in the early gastrula, are expressed in the blastoporal ectoderm in the latter stage as summarized in the first entry in Table 2. Moreover, expression is observed exclusively in the same major domain in the next available stage for at least 69 % of all instances of expression limited to either of these two major domains, as indicated in the last column of Tables 4 and 5. These observations are a strong indication that the central domain differentiates into the later endoderm and that the central and external rings become oral ectoderm. The vegetal hemisphere likely becomes aboral ectoderm, but this is based on a small number of expression patterns (Table 6). These differentiation regions are in agreement with the locations of a dye injected into an *N. vectensis* egg and recorded during embryonic development [11]. Injection of lineage tracers into individual cells of developing *N. vectensis* embryos results in contiguous clones of labeled cells without long distant migration of individual cells (Martindale, unpublished observations). Based on the statistical stability of the main gene expression regions, a regulation mechanism is proposed where genes in early regions activate genes in corresponding later regions, while genes in adjacent regions repress one another (Fig. 21).

If changes in expression patterns occur consistently in many genes, this might indicate a collective cellular motion in the embryo. During gastrulation, expression in the region between the oral and vegetal poles is observed for the quantified profiles of *Anthox1*, *FGFRa*, *FoxD.1*, *Sox1*, *Sox3* and *Rx*. Due to intense *Sox1* expression in the oral pole, the *Sox1* profiles are outside the vegetal ring clusters. *Anthox1*, *FGFRa* and *FoxD.1* are likewise classified as members of the vegetal pole cluster because their strongest expression occurs in the corresponding region. For these six genes, a single *in situ* in the blastula stage has been stored: this *Sox1* image shows staining throughout the animal hemisphere. This hybridization experiment is a weak indication that genes in the body wall ectoderm are first expressed in the external ring or vegetal ring of the blastula. This is basically the null hypothesis; in the absence of any known collective cell movements in the aboral ectoderm during gastrulation, expression in this region has likely remained stationary from the blastula. The body wall has been included in the proposed regulatory interactions among clustered gene expression regions (Fig. 21). The vegetal domains of *Anthox1*, *FGFRa* and *FoxD.1* become restricted to the aboral end in the planula stage, while *Sox1* and *Sox3* move towards the oral pole in the planula. Meanwhile, the *Rx* domain has significantly expanded in the late planula. These changes in gene expression domains may indicate that ectoderm elongation in the planula stage is most pronounced in the initially narrow *Rx* expression domain. Even though this hypothesis is based on few observations, it could be tested with fate mapping experiments. In the frog *Xenopus laevis*, the gene *rax* promotes cell proliferation in developing retinal tissue [17]; *Rx* may similarly induce tissue growth in *N. vectensis*.

Many hierarchical clusters include profiles with strong pairwise correlations to profiles in other clusters. This is caused by the partial overlap of the expression domains that characterize the clusters and by expression of some genes in both regions. The average cutoff value for the main clusters is 0.9; this value is quite high and indicates fuzzy boundaries between many expression clusters. These fuzzy boundaries between expression clusters are likely due to dynamic changes in gene expression. Out of the 25 genes expressed only in the central domain, 3 (12 %) will exhibit major expression changes, or terminate their expression (Table 4, first row). Out of the 10 genes expressed in the central ring or external ring, 1 (10 %) is not expressed in the oral ectoderm (Table 5, first row). This explains why some genes are expressed in multiple clusters at the same stage, and why some genes appear in clusters for different expression domains between developmental stages.

According to the hypothesis that the position of cells in the planula is largely determined by their position in the blastula, the change of gene expression patterns across major regions must be the result of regulatory action. The added percentages for possible major change and first appearance of gene expression in the early gastrula to early planula stages are 45, 48 and 55 % (Table 8). In comparison, these percentages are 37 and 25 % in the early planula to late planula stages. These observations suggest that more dynamic changes in expression occur during gastrulation than in the period that the planula transforms into a polyp. For the study of pattern formation in *N. vectensis*, recording gene expression during the relatively short period of gastrulation should therefore be more informative than monitoring the planula stages. In general, during gastrulation it is decided in which major region(s) the genes are expressed. Detailed expression patterns arise during the planula stages, generally within the bounds of the major regions determined for each gene at the end of gastrulation. The appearance of differential details explains the decrease in correlation among gene expression patterns after gastrulation (Figs. 10, 11, 12, 17, 18 and 19). This loss of correlation could also be caused by the lower number of quantified patterns in the planula stages; with additional gene expression quantifications in these stages of development it could be tested whether the expression domains diverge or whether they form a new set of clusters.

One aspect of gene expression that requires spatial localization, is the determination of the secondary axis in *N. vectensis*. Individual cells appear indistinguishable during the early cleavage cycles until the early blastula starts oscillating at the animal pole [18]. This oscillation stops at the mid blastula transition, when the synchrony of cell divisions is lost and the blastula remains spherical. The spherical blastula symmetry is permanently broken at the onset of gastrulation [19]. During gastrulation, Bmp ligands and antagonists are asymmetrically expressed along the secondary axis [13]. Based on these observations, determination of the secondary axis may coincide with the mid blastula transition. The asynchrony in cell divisions then produces a stochastic perturbation in a morphogen concentration which would define the secondary axis.

The gene expression database contains various genes that are differentially expressed along the secondary axis, although most are not identified in the expression summaries. From the incomplete list of genes included in the expression pattern overview in this study (Additional file 2), various genes involved in secondary axis formation can already be identified. The genes *Anthox7*, *Anthox8b*, *Bmp2/4*, *chordin*, *Gbx*, *Hex*, *Msx*, *Msx2*, *NvHD060*, *Smad1/5* and *Vent1* exhibit noncylindrical expression patterns. Quantification of these patterns requires a two-dimensional or three-dimensional template and eventually a three-dimensional detection method. Especially *Vent1* asymmetric expression in the early gastrula stands out. In various animals, *vent* genes are involved in a Bmp signaling feedback loop [20]. Curiously, the genes *Bmp2/4*, *chordin* and *Smad1/5* in this signaling pathway are still symmetrically expressed in the *N. vectensis* early gastrula (or at least their asymmetry is less pronounced).

The *N. vectensis in situ* hybridization collection contains expression data for less than half of all developmental stages for most genes stored. Despite the sparsity of this dataset, a large-scale analysis results in meaningful insights. Sparse data is common for databases that contain labor intensive measurements, and standardization allows global analysis approaches to be applied to incomplete biological databases. Developmental gene expression databases contain large sets of genes with many regulatory interactions. Our clustering-by-region approach is convenient to select genes from a large set for computational regulatory network modeling.

## Conclusions

Our cluster analysis indicates that early gene expression domains in *N. vectensis* are spatially separated in a stable sequence along the primary body axis. An additional statistical analysis indicates that precise gene expression domains in *N. vectensis* are generally formed by two processes. Genes that are expressed in the blastula appear in broad expression regions. During gastrulation and planula development, the expression domains are refined within the boundaries of these broad regions.

It should be stressed that no additional experiments have been performed for our study. Spatial expression data in a public gene expression database have been quantified and analysed, and these analyses resulted in new hypotheses on cellular migration in *N. vectensis*. Spatial gene expression patterns have been collected in extensive databases for other model animals as well, and a similar computational approach can generate hypotheses about cellular migration before fate maps are available for these animals.

### Availability of supporting data

The quantified spatial gene expression profiles and descriptions of spatial gene expression patterns supporting the results of this article are included within Additional file 1 and Additional file 2, respectively. The original set of *N. vectensis in situ* hybridizations is available in the Kahi Kai repository, http://www.kahikai.org/index.php?content=list_genes&speciesid=1.

## Additional files

Additional file 1:
**MATLAB files of standardized gene expression profiles derived from stored** ***in situ*** **hybridizations.** The profiles are provided both as separate numerical arrays in labeled .mat files and as a two-column array of cells in the file “allarrs.mat”. For the separate arrays, the labels are the filenames; for the array of cells, the labels are the character arrays in the first column. The labels include the gene name, the developmental stage and, if applicable, the sequence number. For example, the file “admp-relatedbla1.mat” contains the variable “profile”, which is a 1×100 numerical array from the first image during the blastula of admp-related. This numerical array is also located in the second column of the 252×2 cell array called “expressiondata” in the file “allarrs.mat”, behind character array “admp-relatedbla1” in the first column. (cle = cleavage, bla = blastula, ega = early gastrula, mga = mid gastrula, lga = late gastrula, epl = early planula, pla = planula, lpl = late planula). The cell array has been converted to comma separated table “expressiontable.txt”, to be processed outside MATLAB and in modified MATLAB releases. The text file has been produced with the script “exportexpression.m” and can be restored to cell array “expressiondata0” in file “allarrs0.mat” with the script “importexpression.m”. (RAR 230 kb) Additional file 2:
**Observed expression domains of genes with** ***in situ*** **hybridizations stored for multiple stages of embryonic development.** The pattern descriptions are ordered in a Microsoft Access database spreadsheet. Shorthand notations: base = base of tentacle; cells = individual cells; syphonoglyph = syphonoglyph side; tuft = apical tuft; wall = body wall. “N/A” means no hybridization image and description are currently provided for this developmental stage. “?” means an entry in the expression summary is provided without a corresponding hybridization image. “none” means no gene expression is observed. “band” means expression is observed in a ring between the oral and aboral ends. “full” means expression is observed in the complete tissue layer. “biradial” means two disconnected expression domains are observed on opposite sides (suggesting a biradially symmetric pattern). “octoradial?” means expression is observed in eight separate domains, possibly suggesting octoradial symmetry. “one side” means expression is present in one side of the embryo, but this side (either the syphonoglyph side or the non-syphonoglyph side) is not indicated. (ACCDB 5268 kb) Additional file 3:
**Subsequent expression domains of genes from an expression database.** Pairs of subsequently available expression domains (third and fourth columns) are ordered in a Microsoft Excel spreadsheet table by their initial and final stages of development (first and second columns, respectively). The percentages (sixth column) are the relative frequencies (from the gene counts in the fifth column) for each pair of expression domains. A change from cylindrical expression pattern symmetry to biradial or bilateral symmetry is indicated in the seventh column with “symmetry break”. Changes of expression over major regions are indicated in the eighth column (appeared = expression is observed only in the second stage, major change = expression moved to another major region, minor change = expression in the second stage is limited to the same major region(s) as in the first stage, none = no expression is observed in both stages, vanished = expression is observed only in the first stage). (XLSX 21 kb)
